# Efficacy of rituximab in refractory polyarteritis nodosa: a case report

**DOI:** 10.11604/pamj.2023.45.92.36496

**Published:** 2023-06-21

**Authors:** Iméne Boukhris, Mohamed Salah Hamdi, Anis Hariz, Meriem Kesentini, Samira Azzabi, Eya Cherif, Ines Kechaou, Lamia Ben Hassine

**Affiliations:** 1 Department of Internal Medicine, Charles Nicolle Hospital, Tunis, Tunisia,; 2 Faculty of Medicine, University of Tunis El Manar, Tunis, Tunisia,; 3 Department of Anatomic Pathology and Cytopathology, Charles Nicolle Hospital, Tunis, Tunisia

**Keywords:** Biological therapy, polyarteritis nodosa, prednisone, skin ulcer, case report

## Abstract

Polyarteritis nodosa (PAN) is a systemic vasculitis affecting medium and small-sized vessels resulting in multiple organ involvement. Refractory PAN requires a different therapeutic approach. We herein report the case of a 42-year-old male presenting a non-virus-related refractory PAN with a favorable outcome on rituximab. He presented significant weight loss, muscle weakness, peripheral axonal neuropathy, and medium-sized cutaneous vessel necrotizing vasculitis. The patient received high-dose corticosteroids and cyclophosphamide with no significant clinical improvement while developing adverse side effects such as hypertension and diabetes. Rituximab was prescribed as an alternative therapy at 1000 mg on day 0 and day 15. This allowed for complete and rapid control of disease activity with regression of cutaneous injury and substantial improvement of neurological symptoms. In conclusion, using chimeric anti-CD20 monoclonal antibodies, such as rituximab, although rarely reported in refractory non-virus-related PAN, may be an effective alternative therapy, as portrayed in our case.

## Introduction

Polyarteritis nodosa (PAN) is a systemic vasculitis defined by the 2012 Chapel Hill Consensus Conference nomenclature of vasculitis as medium-small-sized necrotizing arthritis [1]. Polyarteritis nodosa may be observed in multiple organs such as the skin, testicles, intestines, and nervous system [2]. The skin may also be the unique site of involvement which is recognized as a separate clinical form: cutaneous PAN. While studies on the treatment of PAN are scarce, it usually includes corticosteroids and immunosuppressive therapy, mainly cyclophosphamide, in order to obtain prompt remission [3]. However, relapses and refractory PAN still pose a management challenge. Treatment with chimeric anti-CD20 monoclonal antibodies, such as rituximab, has already demonstrated its efficiency in multiple systemic diseases and may offer superior results regarding refractory PAN [3]. Presented here is the case of a 42-year-old male patient newly diagnosed with PAN with neurological and severe cutaneous involvement.

## Patient and observation

**Patient information:** a 42-year-old male was admitted to our internal medicine department for a three-month history of fever, generalized weakness, joint pain, and important weight loss of 17kg. He also reported weakness and numbness in both upper limbs. He had a medical history of bilateral femoral head avascular necrosis, both requiring a total hip replacement. He denied the use of illicit drugs and had a 10-pack-year smoking history.

**Figure 1:**
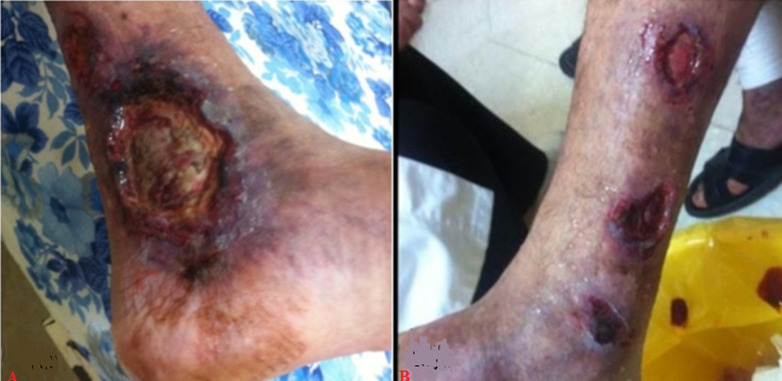
A) extensive necrotic ulcer of the ankle region; B) multiple necrotic ulcers of the lateral side of the leg

**Clinical findings:** on clinical examination, the patient had a fever of 38.7°C. His blood pressure was 130/75 mmHg in both arms. There was palpable purpura with livedo racemosa in both legs. A large necrotic ulcer with a livid wound margin was noted in the ankle region (Figure 1 A). An ophthalmology exam showed conjunctival hyperemia suggesting anterior scleritis. Four days into admission, the patient presented edema of both hands with an extension of ecchymotic lesions to the forearms. We also noted the appearance of multiple necrotic ulcers in the tibial region (Figure 1 B). We objectified predominantly distal muscle weakness associated with diminished deep tendon reflexes. These findings were consistent with peripheral neuropathy confirmed by an electromyogram revealing motor and sensory axonal neuropathy.

**Figure 2:**
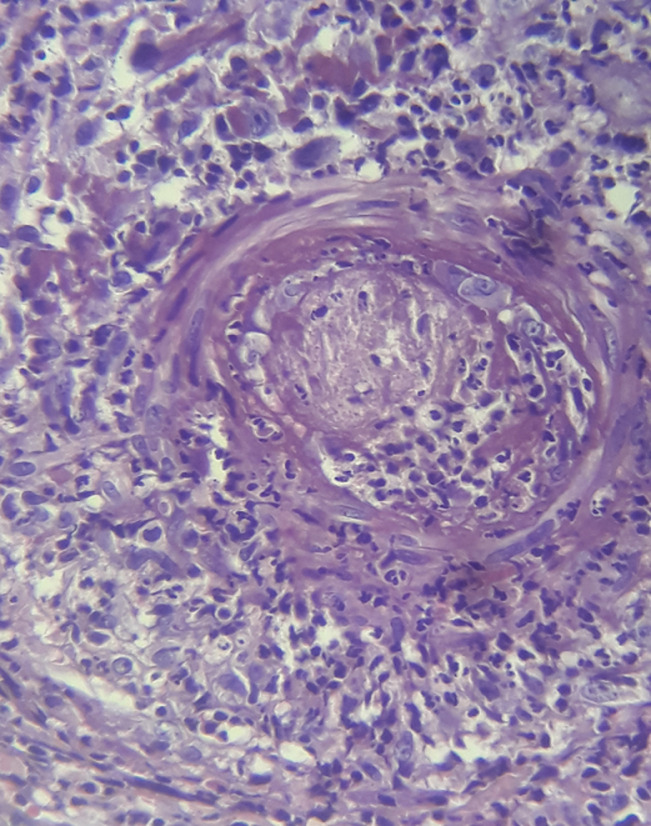
skin biopsy showing medium vessel necrotizing vasculitis at the dermo-hypodermic junction

**Diagnostic assessment:** laboratory routine tests showed increased serum levels of inflammation markers: C-reactive protein at 109 mg/l, erythrocyte sedimentation rate at 60 mm-h1, and fibrinogen serum level at 4.9 g/l (2-4 g/l). The serum protein electrophoresis noted high serum levels of Alpha-2-Globulin and gamma globulin at 11 g/l (5-8 g/l) and 18 g/l (6-11 g/l), respectively. Serum immunoelectrophoresis excluded the presence of monoclonal gammopathy. A complete blood count noted normocytic anemia with hemoglobin of 11.5 g/dl and a mean corpuscular volume of 84.6 fL (80-100 fL). White blood cells and platelet count were within normal range. Vitamin B12, folic acid serum levels, and liver and kidney function tests were within normal range. Chest X-ray was normal. Cardiac ultrasound ruled out infectious endocarditis. Blood cultures were negative. Serum hepatitis B antigen and anti-hepatitis B and C antibodies were not detected. Serologies of HIV, Cytomegalovirus, and Parvovirus B19 were negative. The tuberculin skin test was negative, and the sputum smear was negative for acid-fast bacilli. The immunology work-up did not detect antinuclear antibodies, cryoglobulins, Anti-cyclic citrullinated peptide (anti-CCP), or anti-neutrophil cytoplasmic antibodies. Pathology examination of the purpuric lesion highlighted the presence of medium-vessel necrotizing vasculitis with fibrinoid necrosis of the vessel walls observed at the dermo-hypodermic junction. No involvement of small vessels was noted (Figure 2). An immunofluorescence study was not performed. Contrast-enhanced computed tomography (CT) scan of the abdomen showed no signs of arterial micro-aneurysms. Given the association of weight loss, muscle weakness, peripheral axonal neuropathy, and the findings of cutaneous biopsy, the diagnosis of PAN was established.

**Therapeutic interventions:** treatment was initiated with corticosteroid; the patient received three pulses of methylprednisolone of 1 g each, followed by oral Prednisone of 1 mg/kg daily. Considering the severity of the cutaneous injury and the presence of neurological involvement, the patient was started on monthly pulses of cyclophosphamide at the dose of 0.7g/m^2^ of body surface area. The patient also received Pregabalin at 600 mg/daily. The clinical outcome was unfavorable as the patient's neurological symptoms showed no improvement. We also observed a slight improvement in skin lesions with persistent necrotic and purpuric lesions. The daily steroid dose was upped to 1.5 mg/kg/day with no significant change in clinical presentation. The patient received four pulses of cyclophosphamide (1g each). However, he showed no signs of clinical response to steroid and immunosuppressive therapy. He also presented multiple episodes of skin infection. Given the extension and severity of cutaneous lesions and the persistence of neurological symptoms, we decided to introduce rituximab and discontinue cyclophosphamide. Rituximab was prescribed at the dose of 1000 mg on day 0 and day 15. The daily corticosteroid dose was reduced since the patient had bilateral femoral head avascular necrosis and developed steroid-induced diabetes and hypertension, which comforted our decision to switch therapy.

**Figure 3:**
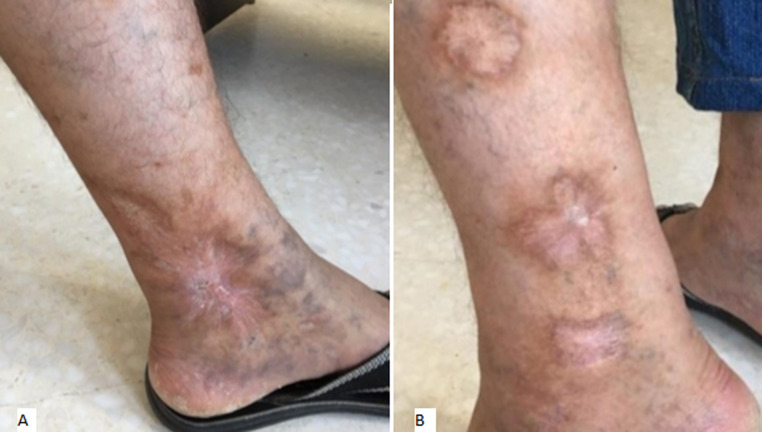
A) complete healing of the skin ulcer of the ankle region; B) favorable outcome of the skin ulcers of the lateral side of the leg

**Follow-up and outcome of interventions:** rapid and complete regression of skin ulcers of the ankle (Figure 3 A) and the tibial (Figure 3 B) region was observed after rituximab administration. As for the neurological involvement, the patient reported persistent mild paresthesia of the left upper limb. A favorable response was obtained on Pregabalin 300 mg/daily. We also observed regression of biological markers of inflammation. After one year of follow-up, the patient continues to be stationary with no clinical or biological signs of PAN relapse while on 5 mg daily of prednisone.

**Patient perspective:** the patient was having a hard time adjusting to the consequence of the uncontrolled disease with the failure of the proposed therapy. Treatment with rituximab was perceived as a last hope which fortunately allowed him to resume his previous lifestyle.

**Informed consent:** informed written consent was obtained from the patient.

## Discussion

We herein report a new case of non-virus-related PAN successfully treated with an anti-CD20+ B- cells antibody, rituximab. The patient presented refractory PAN with peripheral nervous system involvement and severe cutaneous injury. The diagnosis was considered given the clinical presentation associating significant weight loss, muscle weakness, and peripheral axonal neuropathy combined with skin histological findings of medium-sized vessel necrotizing vasculitis. We were unable to obtain a satisfactory clinical response with intravenous cyclophosphamide and had major difficulties with tapering corticosteroids as adverse effects emerged. Once the patient received rituximab, we observed rapid control of PAN activity, and we could safely proceed with tapering corticosteroids. Polyarteritis nodosa is inflammatory necrotizing arthritis, mainly affecting medium-sized blood vessels [1]. General symptoms, mainly fever, are usually present. Cutaneous and nervous involvements are also part of the most frequent symptoms, as observed in our patients. However, other organs, including the urinary tract, digestive system, and lungs, may be involved [2]. It frequently requires concomitant steroid and immunosuppressive therapies to control disease activity [3]. This standard treatment may, in some cases, such as portrayed by our patient, be insufficient to obtain remission. Although the patient received multiple pulses of methylprednisolone coupled with cyclophosphamide, our patient retained severe necrotic cutaneous ulcers with recurrent skin reinfection and disabling neurological involvement. Such severe PAN flare with the refractory outcome on conventional treatments led us to consider other therapeutic options, such as rituximab.

Rituximab is a chimeric monoclonal antibody targeting CD20+ B-cells [4]. It has been proven successful in treating many inflammatory and autoimmune diseases, such as systemic lupus erythematosus, Sjögren´s syndrome, autoimmune cytopenia, and anti-neutrophil cytoplasmic antibodies (ANCA)-associated vasculitis [5,6]. However, to our knowledge, few reports suggest rituximab efficacy in PAN in case of resistance to corticosteroid and classic immunosuppressive therapies [7,8]. The use of rituximab was also reported in isolated cutaneous PAN with satisfying results [9]. Reports similar to ours detailing the efficacy of rituximab in the treatment of refractory PAN are compiled in Table 1 [3,7-11]. However, it is important to note that cases of rituximab resistance in refractory PAN were also reported [12]. Other therapies were prescribed, such as infliximab, with favorable clinical outcomes [13]. Rituximab efficiency in treating vasculitis is well-studied in the case of ANCA-related vasculitis [5]. Such efficacy is underlined by removing pathogenic ANCAs secondary to B-cell depletion [3]. However, B-cells' importance in the pathogenesis of PAN is yet to be fully established, and no antibody has been associated with this disease so far [3]; thus, making use of this B-cell depletion therapy in PAN is questionable. Suppression of pro-inflammatory cytokine by disturbing the interaction between B-cells and T-cells, as well as modifying the T-cells differentiation and the B-cells antigen presentation role, may provide an insight into the mechanism of rituximab efficiency in PAN. Taken together, our report illustrates the difficulties in managing refractory PAN. Our patient, although he did not present any life-threatening involvement, presented refractory cutaneous and neurological injury. He also had multiple adverse effects from corticosteroids. Rituximab was used in this case with satisfactory efficiency and tolerance. As such, rituximab should be considered an alternative therapy in PAN to attain long-term remission and corticosteroid-sparing.

**Table 1:** summarizing recent reports of polyarteritis nodosa treated with rituximab

Author	Pan symptoms	Treatment prior to rituximab	Rituximab protocol	Outcome on rituximab	Relapse and retreatment by rituximab
Seri *et al*.	Cutaneous necrotic ulcer- livedo	Gc, aza, tacrolimus, ivcy	375mg/m^2^/ week x 4	Favorable	Yes
Sonomoto *et al*.	Cutaneous ulcer-mononeuritis multiplex	Gc, ivcy, ivig	375mg/m^2^/ week x 3	Favorable	No
Ribeiro *et al*.	Bilateral orchitis -skin lesions (necrosis, livedo) - acute ischemia of the leg	Gc, ivcy	375mg/m^2^/ week x 4	Partial remission	Yes
Krishnan *et al*.	Cutaneous ulcer	Gc, ivcy	500mg day 0 500mg day 15	Favorable	No
Néel *et al*.	Palpable purpura -mononeuritis multiplex -mesenteric ischemia	Gc	375mg/m^2^/ week x 4	Favorable	No
da Silva *et al*.	Acute ischemia of the leg -cutaneous nodules	Gc, ivcy	1g day 0 1g day 15 1g at six months	Favorable	No

Gc: glucocorticoids, Aza: azathioprine, Ivcy: intravenous cyclophosphamide, Ivig: intravenous immunoglobulin, Mmf: mycophenolate mofetil, Mtx: methotrexate.

## Conclusion

Even without life-threatening involvement, polyarteritis nodosa can still present a management challenge. Although corticosteroid and immunosuppressive therapy are usually sufficient to control disease activity, they may come at the cost of adverse effects, and some patients may require other therapeutic options. As such, while rarely reported in non-virus-related polyarteritis nodosa, rituximab may be considered an effective measure in the case of refractory disease.
